# Timing of menarche and pubertal growth patterns using the QEPS growth model

**DOI:** 10.3389/fped.2024.1438042

**Published:** 2024-08-15

**Authors:** Jenni Gårdstedt-Berghog, Aimon Niklasson, Agneta Sjöberg, A. Stefan Aronson, Aldina Pivodic, Andreas F. M. Nierop, Kerstin Albertsson-Wikland, Anton Holmgren

**Affiliations:** ^1^ Department of Physiology/Endocrinology, Institute of Neuroscience and Physiology, The Sahlgrenska Academy, University of Gothenburg, Gothenburg, Sweden; ^2^ Department of Gynecology and Obstetrics, Halmstad Hospital, Halmstad, Sweden; ^3^ Gothenburg Pediatric Growth Research Center (GP-GRC), Department of Pediatrics, Institute of Clinical Sciences, Sahlgrenska Academy, University of Gothenburg, Gothenburg, Sweden; ^4^ Department of Food and Nutrition and Sport Science, University of Gothenburg, Gothenburg, Sweden; ^5^ APNC Sweden, Gothenburg, Sweden; ^6^ Department of Ophthalmology, Institute of Neuroscience and Physiology, The Sahlgrenska Academy, University of Gothenburg, Gothenburg, Sweden; ^7^ Muvara bv, Multivariate Analysis of Research Data, Leiderdorp, Netherlands; ^8^ Department of Pediatrics, Halmstad Hospital, Halmstad, Sweden; ^9^ Department of Research and Development, Region Halland, Halmstad, Sweden

**Keywords:** menarcheal age, pubertal growth, pubertal timing, menarche, childhood BMI, parental heights, pubertal onset, QEPS growth model

## Abstract

**Objectives:**

To explore the timing of menarche, postmenarcheal growth, and to investigate the impact of various variables on menarcheal age and postmenarcheal and pubertal growth.

**Study Design:**

This longitudinal community population-based study analyzed pubertal growth and menarcheal age in 793 healthy term-born Swedish girls, a subset of the GrowUp_1990_Gothenburg cohort. The timing of menarche and postmenarcheal growth was related to variables from the Quadratic-Exponential-Pubertal-Stop (QEPS) growth model, birth characteristics, and parental height. Multivariable models were constructed for clinical milestones; at birth, age 7 years, pubertal growth onset, and midpuberty.

**Results:**

Menarche aligned with 71.6% (18.8) of the QEPS model's specific pubertal growth function, at a mean age of 13.0 (1.3) years, ranging from 8.2 to 17.2 years. Postmenarcheal growth averaged 8.0 (4.9) cm, varying widely from 0.2 to 31.1 cm, decreasing with later menarche. Significant factors associated with menarcheal age included height at 7 years, childhood body-mass index, parental height, and QEPS-derived pubertal growth variables. Multivariable models demonstrated increasing explanatory power for each milestone, explaining 1% of the variance in menarcheal age at birth, 8% at age 7 years, 44% at onset of pubertal growth, and 45% at midpuberty.

**Conclusions:**

This study underscores the strong link between pubertal growth and age at menarche. Data available at start of puberty explain 44% of the variation in menarcheal age, apparent on average 3.2 years before menarche. In addition, the study shows a previously seldom noticed wide variation in postmenarcheal height gain from 0.2 to 31.1 cm.

## 1 Introduction

Menarche is a pivotal event in female pubertal maturation, signifying a crucial sociocultural transition during adolescence (1). The timing of menarche has a link with various health outcomes and early menarche is associated with overweight in adulthood, cardiovascular risk factors, and breast cancer (2–4). The individual variability of menarcheal age is explained by genetics, body mass index (BMI), ethnicity, and socioeconomic status (2, 5).

Menarche occurs in the latter part of the pubertal period, approximately a year after peak height velocity (PHV), with adult height being attained around 3 years after menarche (5–9). Research focusing on growth patterns around menarche remains limited. Inquiries regarding the interplay between growth and menarche commonly arise in pediatric and adolescent outpatient clinics. The traditional doctrine, as found in textbooks, states that girls grow 5–7.5 cm after menarche; however, limited attention has been given to individual variations in postmenarcheal growth (10–12). Some studies suggest a broader range of variability, with postmenarcheal growth of 5.2–7.5 cm and standard deviation score (SDS) ranging from 2.7 to 10.9 cm (7, 10, 13–15).

**Figure 1:**
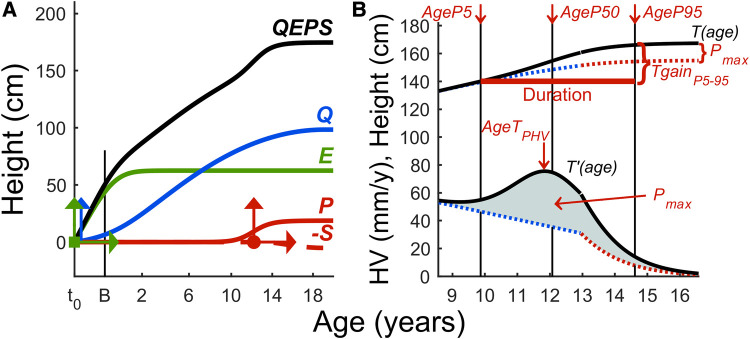
**(A,B)** The QEPS growth model. **(A)** The QEPS model describing individual growth integrates four functions: *Q* and *E* are initiated during early fetal life; *E* plateaus around 2 years of age, while *Q* extends until end of growth (16, 17). A specific pubertal *P*-function adds on the Q during puberty, and growth during puberty is determined by *Q* and *P*. Growth concludes upon activation of *S*. For *E*, *Q*, and *P*, an individual height-scale parameter is defined, and for *E* and *P*, a time-scale parameter; together with *AgeP50* giving six modifying parameters, which enables individual growth curves from birth to adult height. The vertical arrows indicate the individual height-scale parameters and the horizontal time-scale parameters. The individual location of *AgeP50* is marked as a dot. The variable t_0 _= about 6 weeks after conception, and B = birth. Age scale below 3 years is stretched out. **(B)** Total height and height velocity estimated by the QEPS model shown for an individual (16, 17). A*geP*5, *AgeP50*, and *AgeP95* are marked with vertical lines. The duration of puberty is shown by the bold horizontal line. The pubertal height gain is shown from the total growth curve and from the specific *P-*function (*Pmax*).

The Quadratic-Exponential-Pubertal-Stop (QEPS) growth model consists of four growth functions; a basic *Q* growth from early fetal life to end of growth, stopped by an *S*-function, and two specific functions, an exponential *E* for early growth, and an individual specific *P* for pubertal growth, unique in this refinement (Figure 1) (16–20). The combination of this validated model (21, 22) with a healthy growth cohort paves the way for a quantified in-depth analysis of pubertal growth in a novel and detailed manner.

The primary aim was to explore menarcheal age and postmenarcheal growth within the population-based GrowUp_1990_Gothenburg cohort (23). The focus was on assessing growth around menarche and understanding how growth relates to menarche. The secondary aim was to evaluate the association of menarcheal age and postmenarcheal growth with parental height and growth parameters derived from the QEPS model, using multivariable modeling to explain the variation at the following milestones: birth, 7 years of age, pubertal onset, and midpuberty.

## 2 Materials and methods

### 2.1 Study population

The study population was derived from the GrowUp_1990_Gothenburg cohort (23, 24). It comprises individuals born around 1990 in the Gothenburg area and northern Halland county. At inclusion, the mean age of participants was 18.6 years (23). Participants completed a questionnaire covering parental height, country of origin, health status, and age at menarche. Previous growth data were collected from well-baby clinics and school health records.

The inclusion criteria mandated that participants had sufficient longitudinal growth data covering all phases, were born in Sweden, granting access to the Medical Birth Register (MBR), were close to full term at gestational week 36 + 2–41 + 6, had at least one Nordic-born parent, and reported healthy. In total, 918 girls were eligible for the study (24). Lack of information on menarcheal age resulted in exclusion of 125 girls. The final study population consisted of 793 girls. Ethical approval was obtained from the Regional Ethical Review Board in Gothenburg (Ad 444-08). All participants provided informed consent.

### 2.2 Menarcheal data

Participants were asked “When did you have your first period?” with corresponding boxes for year, month, and/or age. Imputations of menarcheal age were performed and internally validated. For girls who wrote year and month (*n* = 285), a calculation was made from birth date to the middle of the recorded month, and when year was recorded (*n* = 38), a similar calculation was modeled. In individuals who wrote year and age (*n* = 103), age was estimated as the remaining part of the written year at the stated age. For girls who mentioned their age (*n* = 367), calculations were made using data of the 184 girls who reported both year, month and age, resulting in reported age + 0.193 years.

### 2.3 Growth data

Information on length, weight, and gestational age at birth was extracted from the MBR and converted to SDS (25). Longitudinal growth data, and height at 7 years calculated by the QEPS model, were transferred to SDS (26) and BMI values to BMI_SDS_ (27). Childhood BMI was estimated using the highest BMI between 3.5 and 7.0 years (BMI_max_). The upper age limit was set to ensure capturing childhood rather than reflecting early puberty. The parameters for pubertal growth were derived from the QEPS model. Calculated adult height was derived from the QEPS model (*T_max_*), and if the measured height exceeded *T_max_,* it was utilized and transferred to SDS using this dataset (24).

Maternal height was sourced from the MBR, supplemented by the questionnaire when missing, while paternal height was sourced from the questionnaire, and transferred to SDS using this dataset. Midparental height (MPH) was calculated as (mother's height_SDS_ + father's height_SDS_)/2. The girl's height in relation to her parents' height (*DiffH-MPH_SDS_*) was calculated as height in SDS at each milestone, minus MPH in SDS.

The variables were separated into those available at clinical milestones for the multivariable modeling analyses. Parental height and birth data comprised the *birth* milestone; childhood growth data up to 7 years were added to the *childhood 7 years of age* milestone; the parameters of pubertal growth onset were added for the *pubertal onset* milestone; and the variables at midpuberty were added for the *midpuberty* milestone (28).

### 2.4 The QEPS model

The QEPS model enables the modeling of individual growth curves (Figure 1) (16, 17). Estimates for pubertal growth are separated into two parts: the specific *P*-function and the basic *QES*-function, together giving the total *T*-function growth during puberty. The estimates were as follows:

- From *the total growth curve, T-function*: age at PHV (*AgeT_PHV_*), total height gain during puberty (*Tpubgain*) and estimated adult height (*T_max_*).
- From the *specific pubertal growth curve, P-function*: onset of pubertal growth defined as 5% of the *P*-function (*AgeP5*), midpuberty as 50% and age at PHV (*AgeP50, AgeP_PHV_*), end of pubertal growth as 95% or 100% (*AgeP95, AgeP100*), duration of the specific *P*-function, growth defined as height gain during AgeP5–AgeP100 (*Ppubgain*), and total specific pubertal height gain (*P_max_*).^18^
- From *the QES-function*: growth during pubertal growth defined by the basic growth (*QESpubgain*).

The percentage of achieved specific pubertal growth at time of menarche (*P%*) was calculated for each individual. Height at menarche was also estimated by the *T*-function at menarcheal age, *T_AgeMenarche_*. The estimates of height gain during puberty were compared calculating *P*-function minus *QES*-function (*Deltapubgain*).

### 2.5 Statistical analysis

Matlab was used to construct longitudinal growth curves for each individual (29). Subsequent computer processing and analyses were carried out using IBM SPSS and SAS (30, 31). Continuous variables were presented as mean, SD, minimum, and maximum values.

The primary outcome was age at menarche. The secondary outcome was postmenarcheal growth from menarche until adult height. Associations between selected explanatory variables, reported before or around the time of the respective outcome, and outcome variables were explored through univariable linear regression models. A significance level of *p* < 0.05 following the Bonferroni–Holm adjustment was considered statistically significant. Multivariable linear regression models were applied to variables separately for the known clinical milestones: birth, childhood at age 7 years, puberty onset, and midpuberty, aiming to mitigate the issue of anticipated multicollinearity. From each milestone, the statistically independent variables were selected for evaluation in the final multivariable model. Variable selection was defined using stepwise forward and backward selection.

Both association variables and outcome variables were standardized for their own SD in the cohort, in order to be able to compare standardized beta-coefficients for the association variables between and within outcome variables. Along with the standardized-beta estimates, 95% confidence intervals (CI) were presented, with *p*-values, R^2^, and for the multivariable models, partial R^2^. Correlation was assessed by Pearson correlation-coefficient.

## 3 Results

The characteristics for all study variables including QEPS are presented in Table 1, where the important clinical data are highlighted. The non-response analyses are presented in Table 2.

**Table 1:** Descriptive data for study variables and outcomes at each milestone.

	Variable	Mean (standard deviation) median (range), *n* = 793
Parental heights	Motheŕs height (Mheight) cm	167.19 (6.01) 167.00 (150.00–188.00), *n* = 776
	Motheŕs height (Mheight) SDS	−0.00 (1.00) −0.03 (−2.86–3.46), *n* = 776
	Fatheŕs height (Fheight) cm	181.41 (6.66) 182.00 (161.00–199.00), *n* = 751
	Fatheŕs height (Fheight) SDS	0.00 (1.00) 0.09 (−3.06 to 2.64), *n* = 751
	Midparental height (*MPH_SDS_*) SDS	−0.00 (0.78) −0.03 (−2.66 to 2.74), *n* = 751
Birth	Gestational age (GA) weeks, calculation from days to weeks = x7 + 3.5	40.15 (1.32) 40.29 (36.29–42.86)
	Birth length (BL) cm	50.09 (2.05) 50.00 (42.00–56.00)
	Birth length (BL) SDS	−0.53 (1.22) −0.57 (−4.60 to 3.67)
	Height at birth in relation to MPH (*DiffH-MPH_SDS_*) SDS	−0.51 (1.22) −0.55 (−4.64 to 3.28), *n* = 751
	Birth weight (BW) kg	3.53 (0.50) 3.51 (1.85–5.43)
	Birth weight (BW) SDS	−0.20 (1.07) −0.16 (−4.87 to 3.27)
Childhood 7 years	*E* –function timescale ( *Etsc* )	0.99 (0.09) 1.00 (0.74–1.44)
	Gain in adult height due to *E*–function growth (*E_max_*) cm	62.76 (2.83) 62.63 (55.40–73.48)
	Gain in adult height due to *E*–function growth (*E_max_*) SDS	−0.02 (1.00) −0.07 (−2.62–3.76)
	Gain in adult height due to *Q*–function growth (Q_max_) cm	98.51 (7.83) 98.45 (72.52–124.95)
	Gain in adult height due to *Q*–function growth (Q_max_) SDS)	−0.02 (1.00) −0.03 (−3.34 to 3.36)
	Max BMI 3.5–7 years (*BMI_max_)* kg/m^2	16.25 (1.54) 16.03 (12.29–22.72)
	Max BMI 3.5–7 years (*BMI_max_)* SDS	0.38 (1.01) 0.35 (−3.25 to 3.63)
	Height (*T_7y_*) cm	124.29 (4.94) 124.20 (110.01–143.20)
	Height (*T_7y_*) SDS	−0.03 (0.99) −0.05 (−2.91 to 3.78)
	Height at 7 years in relation to MPH (*DiffH-MPH_SDS_*) SDS	−0.01 (0.87) −0.01 (−2.41 to 2.81), *n* = 751
Pubertal onset	**Height at 5% of *P*-function ( *T_AgeP5_* ) cm**	**139.57 (7.04) 139.07 (120.36–163.81)**
	Height at 5% of *P*-function ( *T_AgeP5_* ) SDS	−0.15 (0.99) −0.18 (−3.14 to 3.70)
	Height at 5% of *P*-function in relation to MPH (*DiffH-MPH_SDS_*) SDS	−0.13 (0.91) −0.16 (−2.51 to 2.84), *n* = 751
	**Age at 5% of *P*-function (*AgeP5*) years**	**9.76 (1.00) 9.78 (5.95–12.93)**
Midpuberty	Height at 50% of *T*-function ( *T_AgeP50_* ) cm	154.62 (6.56) 154.41 (135.61–178.05)
	Height at 50% of *T*-function ( *T_AgeP50_* ) SDS	0.03 (0.99) 0.02 (−2.92 to 3.42)
	Height at *AgeP50* in relation to MPH ( *DiffH-MPH_SDS_* ) SDS	0.05 (0.94) 0.01 (−2.34 to 2.93), *n* = 751
	Age at 50% of *P*-function (*AgeP50*) years	11.98 (1.00) 12.00 (8.25–15.14)
	Menarche before *AgeP50*	109 (13.7%)
	Age at PHV of *P*-function (*AgeP_PHV_*) years	11.91 (1.00) 11.92 (8.17–15.07)
	Menarche before *AgeP_PHV_*	97 (12.2%)
	Age at PHV of *T-*function (*AgeT_PHV_*) years	11.74 (1.01) 11.76 (7.70–14.84), *n* = 788
	**Duration from *AgeP5* to menarche, years**	**3.20 (1.00) 3.13 (−0.67 to 7.18)**
	**Duration from *AgeP_PHV_* to menarche, *years***	**1.05 (0.99) 0.99 (−2.81 to 5.04)**
	*P* -function at menarche (*P%*)	71.56 (18.83) 75.02 (1.97–99.50)
	**Height at age of menarche (*T_AgeMenarche_)* cm**	**160.18 (8.00) 160.40 (132.43–186.41)**
End-puberty	Height at *AgeP95* ( *T_AgeP95_* ) cm	166.33 (6.46) 166.16 (147.41–188.53)
	Age at 95% of *P*-function (*AgeP95*) years	14.54 (1.00) 14.54 (10.90–17.69)
	**Duration from menarche to *P95,* years**	**1.59 (0.98) 1.64 (−2.41 to 5.44)**
	**Duration from menarche to *P99*, years**	**3.24 (0.98) 3.28 (−0.76 to 7.10)**
	Total *P*-function growth (*P_max_*) cm	12.92 (3.58) 12.96 (0.00–23.43)
	Total *P*-function growth (*P_max_*) SDS	−0.01 (1.01) −0.00 (−3.66 to 2.95)
	Total height gain during *P5-P100* (*Tpubgain*) cm	28.25 (3.85) 28.12 (18.22–42.20)
	Height gain from *P*-function during *P5-P100* (*Ppubgain*) cm	12.28 (3.40) 12.31 (0.00–22.26)
	Height gain from *QES*-function during *P5-P100* (*QESpubgain*) cm	15.97 (2.77) 15.79 (9.36–28.46)
	*Ppubgain-QESpubgain* (*Deltapubgain*) cm	−3.70 (4.86) −3.39 (−25.47 to 10.15)
Adult height	Adult heigh, cm	168.12 (6.48) 167.94 (150.17–189.88)
	Adult height, SDS	−0.00 (1.00) −0.03 (−2.77 to 3.35)
	Adult height in relation to MPH. ( *DiffH-MPH_SDS_* ) SDS	0.02 (0.75) 0.02 (−2.23 to 2.21), *n* = 751
Study outcomes	**Postmenarcheal height gain (*Tgain_AgeMenarche_* *_−AH_* ) cm**	**7.95 (4.95) 7.07 (0.19–31.14)**
	**Age at menarche (AgeMenarche) years**	**12.96(1.32) 13.17 (8.20–17.21)**

The most important results are highlighted in bold.

**Table 2:** Descriptive data for study variables for included and excluded subjects. Mann–Whitney *U*-test was used to compare between the included and excluded individuals.

	Variable	Mean (standard deviation) median (range)		
		Included, *n* = 793	Excluded, *n* = 125	*p*-Value
Childhood 7 years	*E* –function timescale ( *Etsc* )	0.99 (0.09) 1.00 (0.74–1.44)	1.00 (0.09) 0.99 (0.81–1.25)	0.68
	Gain in adult height due to *E*–function growth (*E_max_*) cm	62.76 (2.83) 62.63 (55.40–73.48)	63.06 (3.02) 62.63 (56.20–71.11)	0.44
	Gain in adult height due to *E*–function growth (*E_max_*) SDS	−0.02 (1.00) −0.07 (−2.62 to 3.76)	0.09 (1.06) −0.07 (−2.34–2.92)	0.44
	Gain in adult height due to *Q*–function growth (Q_max_) cm	98.51 (7.83) 98.45 (72.52–124.95)	97.91 (7.90) 97.72 (74.38–120.87)	0.52
	Gain in adult height due to *Q*–function growth (Q_max_) SDS	−0.02 (1.00) −0.03 (−3.34 to 3.36)	−0.10 (1.01) −0.12 (−3.10 to 2.84)	0.52
	Height (*T_7y_*) cm	124.29 (4.94) 124.20 (110.01–143.20)	124.22 (5.12) 124.80 (109.00–142.39)	0.96
	Height (*T_7y_*) SDS	−0.03 (0.99) −0.05 (−2.91 to 3.78)	−0.05 (1.03) 0.07 (−3.11 to 3.61)	0.96
Pubertal onset	Height at 5% of *P*-function ( *T_AgeP5_* ) cm	139.57 (7.04) 139.07 (120.36–163.81)	140.21 (6.80) 139.19 (124.96–160.32)	0.32
	Height at 5% of *P*-function ( *T_AgeP5_* ) SDS	−0.15 (0.99) −0.18 (−3.14 to 3.70)	−0.20 (1.02) −0.11 (−3.24 to 3.35)	0.78
	Age at 5% of *P*-function (*AgeP5*) years	9.76 (1.00) 9.78 (5.95–12.93)	9.92 (0.93) 9.82 (7.97–12.54)	0.16
Midpuberty	Height at 50% of *T*-function ( *T_AgeP50_* ) cm	154.62 (6.56) 154.41 (135.61–178.05)	155.26 (6.07) 155.15 (141.02–173.46)	0.25
	Height at 50% of *T*-function ( *T_AgeP50_* ) SDS	0.03 (0.99) 0.02 (−2.92 to 3.42)	−0.02 (1.00) −0.04 (−2.66 to 2.80)	0.65
	Age at 50% of *P*-function (*AgeP50*) years	11.98 (1.00) 12.00 (8.25–15.14)	12.15 (0.93) 12.08 (10.25–14.77)	0.14
	Age at PHV of *P*-function (*AgeP_PHV_*) years	11.91 (1.00) 11.92 (8.17–15.07)	12.07 (0.93) 12.01 (10.17–14.69)	0.15
	Age at PHV of *T*-function (*AgeT_PHV_*) years	11.74 ± 1.01 11.76 (7.70–14.84) *n* = 788	11.90 ± 0.93 11.87 (10.07–14.48) *n* = 125	0.15
	Duration from *AgeP_PHV_* to menarche, years	1.05 (0.99) 0.99 (−2.81 to 5.04)		
	*P* -function at menarche (*P%*)	71.56 (18.83) 75.02 (1.97–99.50)		
	Height at age of menarche (*T_AgeMenarche_*) cm	160.18 (8.00) 160.40 (132.43–186.41)		
End-puberty	Height at *AgeP95* ( *T_AgeP95_* ) cm	166.33 (6.46) 166.16 (147.41–188.53)	167.02 (5.76) 166.58 (153.83–182.75)	0.22
	Age at 95% of *P*-function (*AgeP95*) years	14.54 (1.00) 14.54 (10.90–17.69)	14.72 ± (0.93) 14.64 (12.76–17.34)	0.13
	Duration from menarche to *P95,* years	1.59 (0.98) 1.64 (−2.41 to 5.44)		
	Duration from menarche to *P99,* years	3.24 (0.98) 3.28 (−0.76 to 7.10)		
	Total *P*-function growth (*P_max_*) cm	12.92 (3.58) 12.96 (0.00–23.43)	13.40 (3.60) 13.14 (4.69–26.85)	0.20
	Total *P*-function growth (*P_max_*) SDS	−0.01 (1.01) −0.00 (−3.66 to 2.95)	0.12 (1.02) 0.05 (−2.34 to 3.92)	0.20
	Total height gain during *P5-P100* (*Tpubgain*) cm	28.25 (3.85) 28.12 (18.22–42.20)	28.28 (4.00) 28.27 (18.00–40.25)	0.92
	Height gain from *P*-function during *P5-P100* (*Ppubgain*) cm	12.28 (3.40) 12.31 (0.00–22.26)	12.73 (3.42) 12.48 (4.46–25.51)	0.20
	Height gain from *QES*-function during *P5-P100* (*QESpubgain*) cm	15.97 (2.77) 15.79 (9.36–28.46)	15.55 (2.60) 15.50 (9.74–21.44)	0.16
	*Ppubgain-QESpubgain* (*Deltapubgain) cm*	−3.70 (4.86) −3.39 (−25.47 to 10.15)	−2.82 (4.58) −2.34 (−14.80 to 15.77)	0.06
Adult height	Adult height, cm	168.12 (6.48) 167.94 (150.17–189.88)	168.81 (5.78) 167.95 (155.50–184.04)	0.22
Study outcomes	Postmenarcheal height gain (*Tgain_AgeMenarche_* *_−AH_* ) cm	7.95 (4.95) 7.07 (0.19–31.14)		
	Age at menarche (*AgeMenarche*) years	12.96(1.32) 13.17 (8.20–17.21)		

### 3.1 Descriptives of menarcheal age and postmenarcheal growth

**Figure 2:**
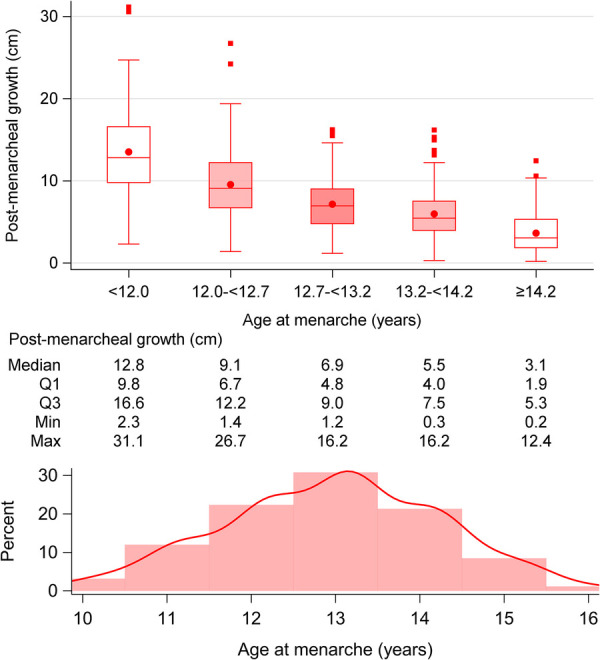
Age at menarche in relation to postmenarcheal growth: postmenarcheal growth in cm, divided into five quintiles of menarcheal age:<12, 12–<12.7, 12.7–<13.2, 13.2–<14.2, and ≥14.2 years. The postmenarcheal height gain decreases with older age at menarche. A histogram showing the distribution of menarcheal age is also presented.

**Figure 3:**
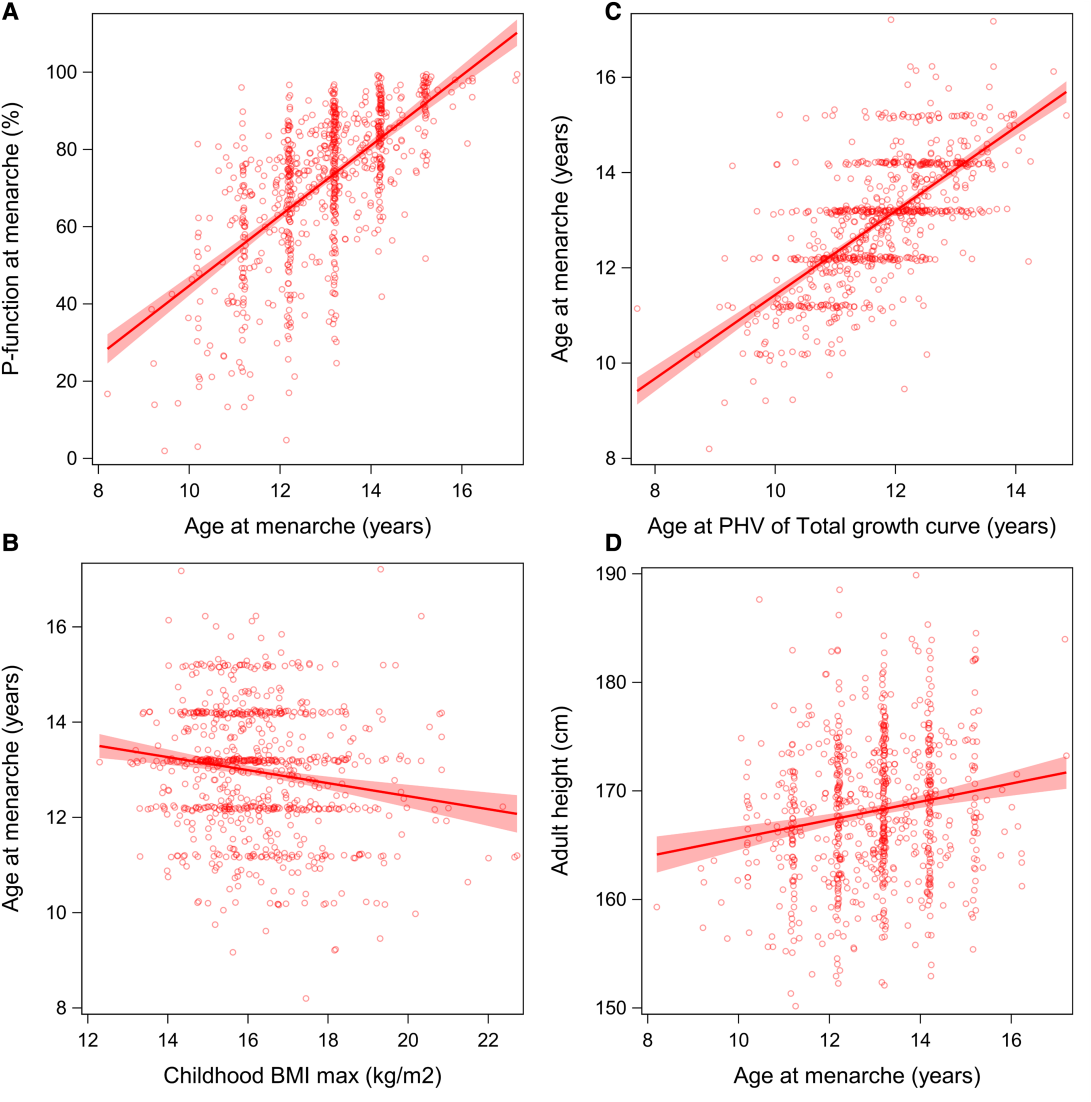
**(A–D)** Menarcheal age in relation to *P%*, BMI, *AgeT_PHV_*, and adult height: Associations between menarcheal age and *P%*, BMI_max_, adult height, and *AgeT_PHV_*. **(A**, **C)** A positive association between menarcheal age and *P%* and *AgeT_PHV_*. **(B)** A negative correlation with BMI_max_. **(D)** Greater adult height is associated with later menarche.

Mean menarcheal age was 13.0 (1.3) years, ranging from 8.2 to 17.2 years (Figure 2). Postmenarcheal height gain averaged 8.0 (4.9) cm, ranging from 0.2 to 31.1 cm. Postmenarcheal growth correlated significantly with total height gain during puberty (R^2^ 0.014). Menarcheal age aligned with the achievement of 71.6% (18.8) of the P-function, and older age was associated with a higher *P%,* illustrated in Figure 3. Menarche occurred at a mean height of 160.2 (8.0) cm. On average, menarche occurred 3.2 (1.0) years after onset of pubertal growth. The duration of pubertal growth after menarche was 1.6 (1.0) years. For the majority of the girls, menarche occurred after *AgeP_PHV_*, for 12%, menarche occurred before.

### 3.2 Linear regression models for menarcheal age

**Figure 4:**
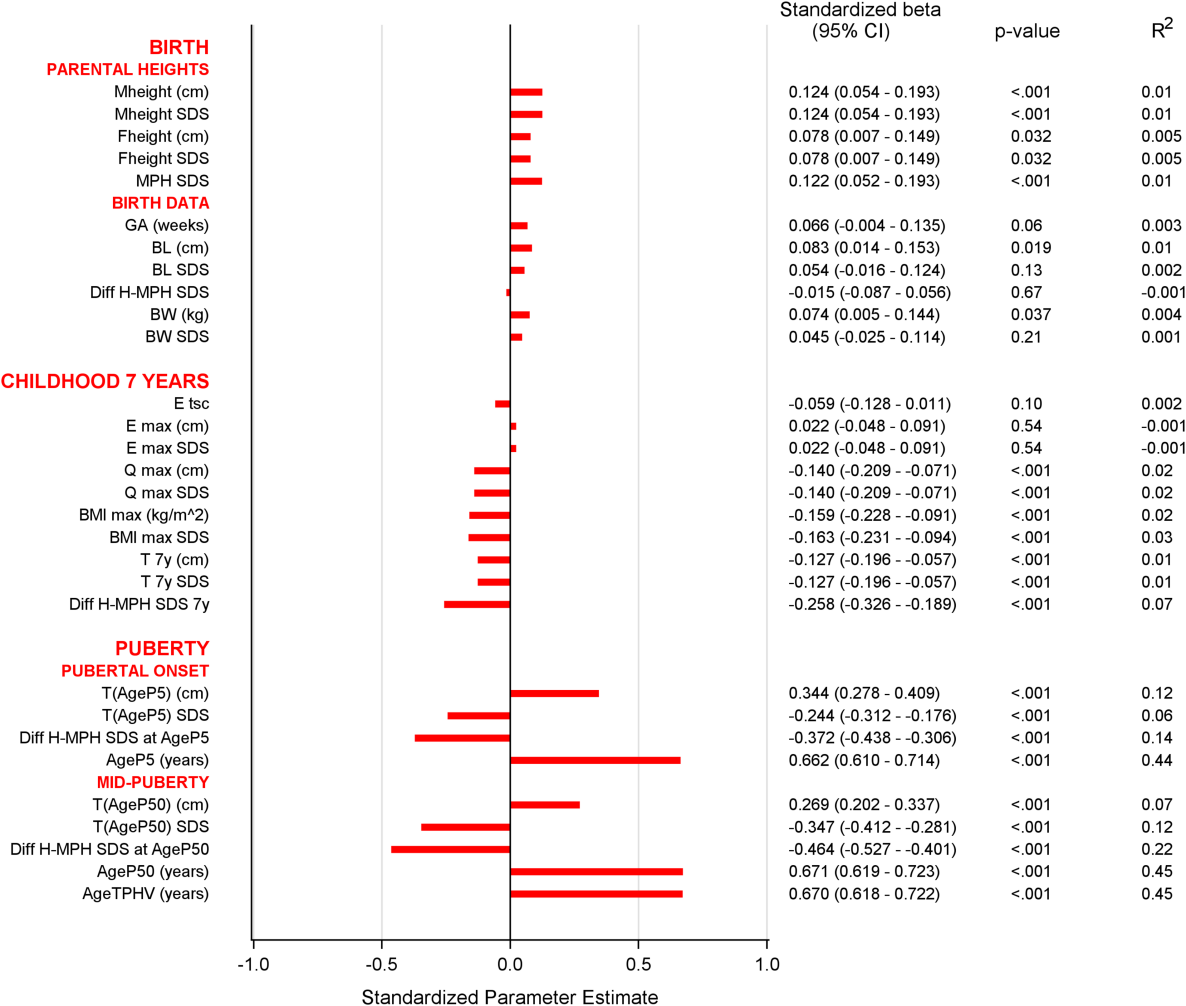
Univariable analyses of menarcheal age at each milestone: Bar graph of univariable analyses of menarcheal age. The variables shown to be significantly associated to menarche were mother's height, MPH, BMI_max_, childhood height, and all variables of pubertal growth derived by the QEPS model. For abbreviations, see Table 2.

**Figure 5:**
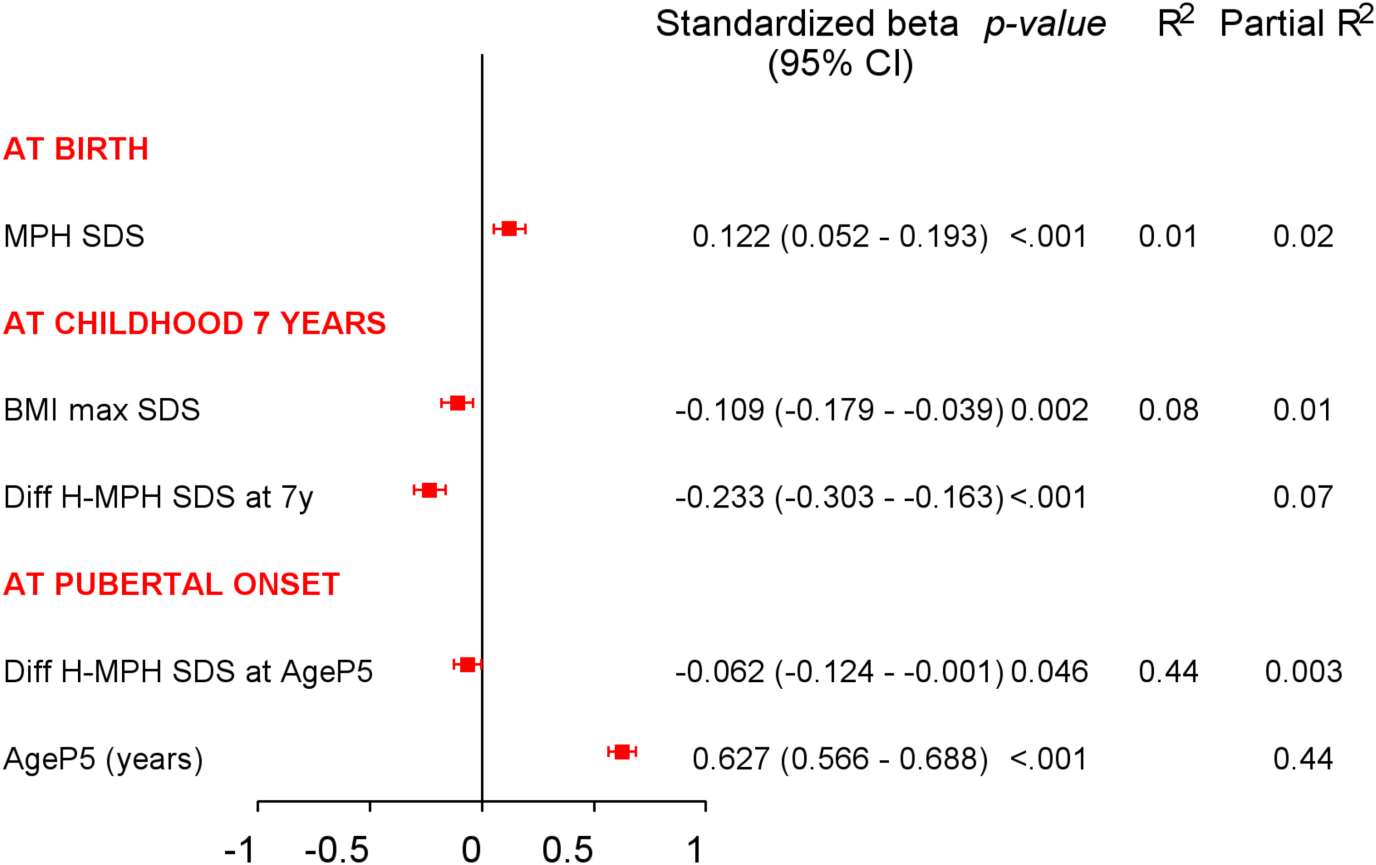
Multivariable analyses of menarcheal age at each milestone: multivariable linear analyses with variables known at clinical milestones: birth, childhood at 7 years, and pubertal growth onset. *R*^2^-values show increased explanation in the variation of menarcheal age, which was 1% at birth, 8% at childhood 7 years, and 44% at pubertal onset. Partial *R*^2^-values present explanation of each variable separately.

**Figure 6:**
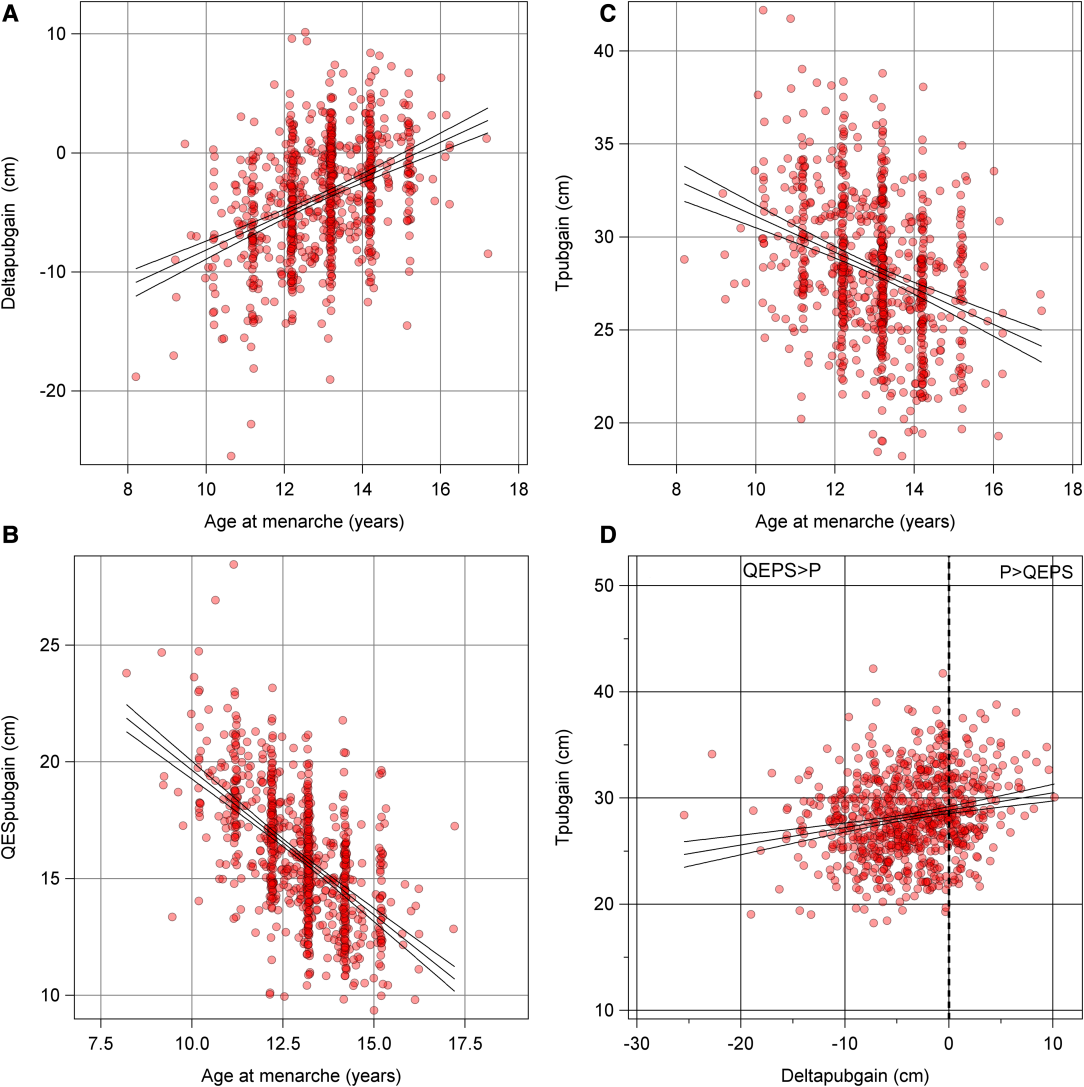
**(A–D)** Total pubertal height gain and *Deltapubgain*: Graphs presenting total pubertal height gain, and the two estimates of pubertal growth of the QEPS model; the specific *Ppubgain* and basic *QESpubgain*, in relation to menarche*.* **(A)** An older menarcheal age associates to a larger contribution of *P* in growth. Basic *QESpubgain* ceases with older menarcheal age, as shown in **(B)**. **(C)** Decreasing total height gain during puberty with older menarcheal age. **(D)** The relationship between total pubertal gain and *Deltapubgain*; a high total height gain is associated with a larger contribution of *Ppubgain.*

Univariable analyses for age at menarche are presented in Figure 4. Multivariable models explaining the variation in menarcheal age showed increasing *R*^2^ values for each milestone, from 1% at birth, 8% at age 7 years, 44% at puberty onset, to 45% at midpuberty (Figure 5). Total growth, *Tpubgain*, was negatively correlated to age at menarche (*R*^2^ 0.1, *p* < 0.001). The specific *Ppubgain* was higher in girls with early menarche and contributed more to total growth relative to the basic *QESpubgain*. These associations are shown in Figure 6.

*At birth,* in univariable analyses, motheŕs height showed a positive correlation to age at menarche. MPH in SDS demonstrated an association between taller parents and later menarche. This was the only one independently significant variable selected from the multivariable regression model for menarcheal age, explaining 1% of the variability of menarcheal age [standardized-B 0.12 (95% CI 0.05–0.19), *p* < 0.001, *R*^2^ 0.01].

*At 7 years of age,* in univariable analyses, taller height was associated with earlier menarche as well as high BMI_max_ presented in Figure 3. *DiffH-MPH_SDS_* and BMI_max_ were shown to be independent significant explanatory variables, yielding a combined explanation of 8%. (*R*^2^ 0.08; *DiffH-MPH_SDS_*: standardized-B −0.23 (95% CI −0.30 to −0.16), *p* < 0.001, partial *R*^2^ 0.07; BMI_max_ in SDS: standardized-B −0.11 (95% CI −0.18 to −0.04), *p* 0.002, partial *R*^2^ 0.01).

*At pubertal growth onset,* in univariable analyses, both age and height exhibited associations to menarcheal age, with older age and taller height associated with later menarche. In multivariable models, *AgeP5* and *DiffH-MPH_SDS_* emerged as independent significant explanatory variables in relation to age at menarche. Together, they explained 44% of the variation in menarcheal age, with *AgeP5* being the most influential variable (partial *R*^2^ 0.44).

*At midpuberty,* in univariable analyses, all QEPS variables demonstrated association with age at menarche, explaining variance ranging from 7% to 45%. The strongest association was seen with the variables *AgeP50* and *AgeT_PHV_* (Figure 4). On average, menarche occurred 1.23 (0.99) years after *AgeT_PHV_* and a linear association was observed, as seen in Figure 3. Similar results were shown for *AgeP50*, with menarche occurring 0.97 years after *AgeP50*.

### 3.3 Associations between menarcheal age and adult height

Individuals with taller adult height were older at menarche (Figure 3). For each year of later pubertal growth adult height increased by 1.6 cm, and for each year of later menarche, individuals became approximately 0.84 cm taller. Using *DiffH-MPH_SDS_* at menarche, results showed that individuals were estimated to be taller than their MPH when menarche occurred later than 12.6 years of age.

### 3.4 Linear regression models for postmenarcheal height gain

**Figure 7:**
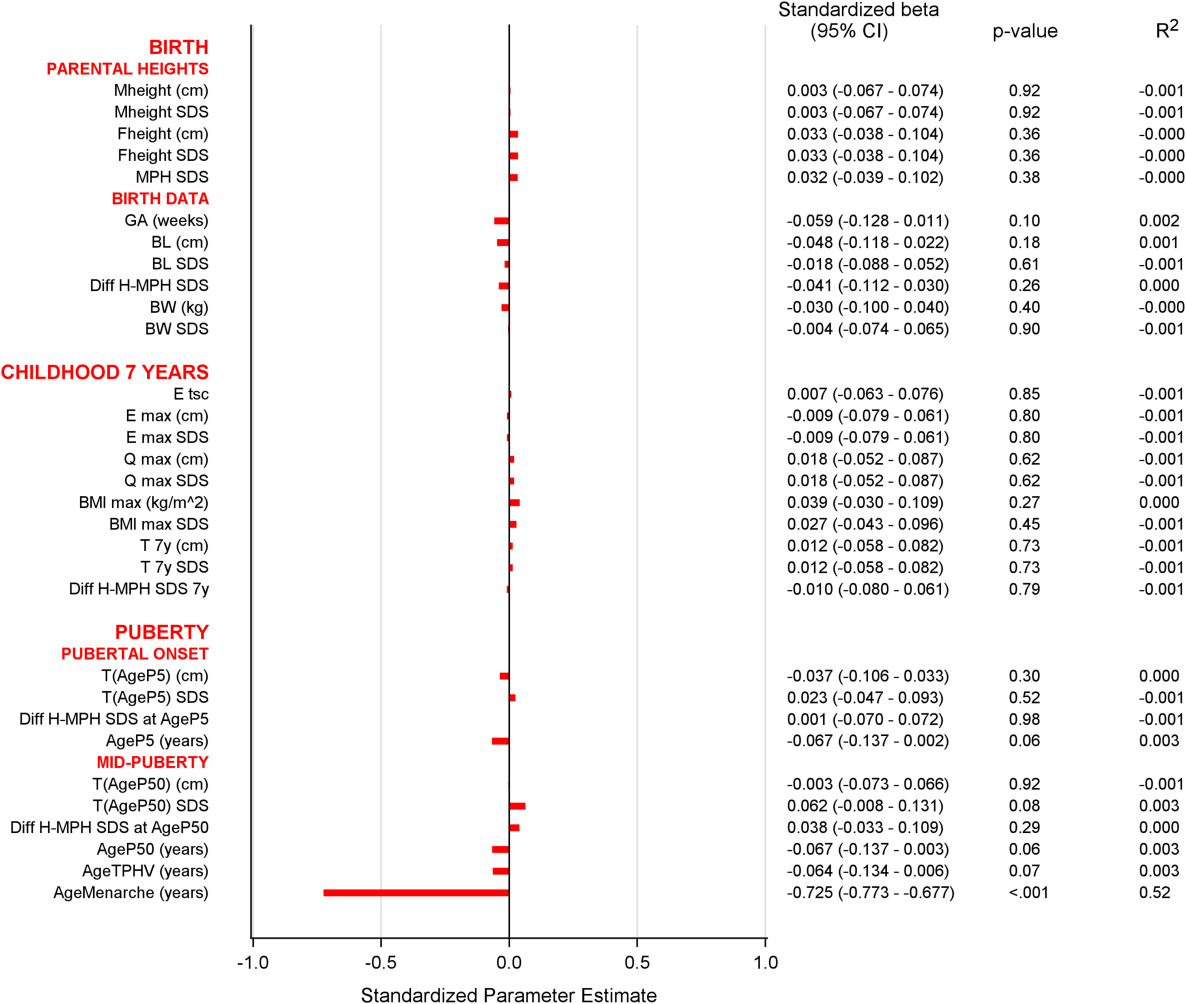
Univariable analyses of postmenarcheal growth at each milestone: Bar graph of univariable analyses of postmenarcheal growth. A significant association with age at menarche was seen as also shown in Figure 2. No other variables showed significant association with postmenarcheal growth. For abbreviations, see Table 2.

No significant associations were observed between postmenarcheal height gain and variables of birth, childhood height and BMI, puberty, or parental height (Figure 7). The height gain was negatively associated with age at menarche (Figure 2). Postmenarcheal growth was significantly associated with *Tpubgain*. Given that the only significantly associated variables were menarcheal age and *Tpubgain*, no multivariable analyses were performed.

## 4 Discussion

This study investigates total growth in relation to timing of menarche in a Swedish population-based cohort. The QEPS-model was used for growth analysis with the emphasis on puberty. Menarcheal age aligned largely with the QEPS-model's specific pubertal growth function and parental height. Mean menarcheal age was 13.0 (1.3) years, ranging from 8.2 to 17.2 years. A notable clinically important outcome of this study is the detailed exploration of postmenarcheal growth; our study shows a mean growth of 8.0 (4.9) cm and unveils a substantial variability contrary to the prevalent assumption, ranging from 0.2 to 31.1 cm.

In multivariable models, the explained variation in menarcheal age between variables at each milestone increased progressively. At birth, parental height explained only 1% of the variation in menarcheal age. At 7 years of age, height and BMI accounted for 8% of the variation. At onset of pubertal growth, information at this point accounted for 44% of the variation in menarcheal age. The degree of association did not improve later in puberty, as analysis at midpuberty exhibited a similar 45% association. The results underscore the strong association between pubertal growth and menarcheal age.

The mean menarcheal age of 13.0 (1.3) years aligns with earlier knowledge and marks a cessation of secular trends of menarcheal age (32). Age at menarche ranged from 8.2 to 17.2 years, which is of clinical importance considering the large individual variation. There was a significant correlation between age at menarche, childhood height and BMI, mother's height, and variables of pubertal growth, which emphasizes the multifactorial nature of menarche. Systems for height prediction may include menarcheal age. The residuals in the Tanner–Whitehouse height prediction (TW-2) are reduced by over 50% after menarche, which emphasizes the physiological importance of the menarcheal landmark in the growth process (33).

We demonstrated that menarcheal age had an inverse relationship with BMI during childhood, and with total height gain during puberty, confirming earlier evidence (3, 10, 34). Childhood height exhibited a stronger correlation with menarcheal age than previously known (3). This underscores the significance of height during childhood as an indicator for age at menarche. Birth characteristics and gestational age in our study were not significantly associated with menarcheal age, an area where earlier research has shown conflicting results (3, 5, 34, 35).

Maternal height had a significant association with both age at menarche and adult height, and was the basis of the association between MPH and menarcheal age, showing that taller parents tend to have daughters experiencing later menarche and reaching taller adult height. Girls experiencing later menarche reached taller adult height and were older at midpuberty. This affirms knowledge on the correlation between menarche and adult height (36), and in cases of extreme delays in puberty, an increase in adult height has been described, as well as shorter adult height in *pubertas praecox* (37, 38).

Menarche occurred, on average, when 71% of specific pubertal growth was achieved. The correlation between menarche and pubertal growth was evident, with menarche occurring about 1 year after *AgeP50* and 1.2 years after *AgeT_PHV_*, in accordance with previous research (39, 40). It is noteworthy that in our findings 12% of the girls experienced menarche before *P_PHV_*. The finding may imply a looser relation between midpubertal growth and menarche (9, 40). Pubertal growth extended 1.6 years after menarche, a duration shorter than observed in previous studies, potentially attributed to our choice of the *AgeP95* definition for the conclusion of puberty. This was chosen based on the understanding that growth diminishes after reaching 95% of the pubertal growth aiming for narrow CI and therefore is a more precise measure of puberty duration (16). The duration of pubertal growth after menarche when calculating the end of puberty as 99% of the *P*-function was 3.2 years (Table 2).

This study revealed that the specific pubertal height gain was independent of age at menarche, contrary to the total height gain observed during puberty. In girls with high total growth during puberty, the *Deltapubgain* was high, meaning these girls had a higher portion of growth derived from the *P*-function (Figure 6). However, greater total height gain in girls with early menarche was attributed to a high ongoing basic *QES*-growth. Girls with later menarche had growth dominated by specific *P-*growth and thereby high *Deltapubgain*, but less growth in total due to a ceasing *QES*-function growth (Figure 6) (17).

A clinically important outcome of this study is the detailed exploration of postmenarcheal growth, a topic that has received limited attention in previous research. Contrary to the general understanding of 5–7.5 cm growth postmenarche, our study shows a mean growth of 8.0 (4.9) cm, and unveils a substantial variability in this pattern, ranging from 0.2 to 31.1 cm. The individual variations in postmenarcheal growth highlight the inadequacy of a one-size-fits-all approach and emphasize the need for personalized growth trajectories. Postmenarcheal height gain was strongly inversely correlated with age at menarche, indicating a higher gain in girls with early menarche, in line with previous research (9, 10, 41).

In this study, participants recalled information about menarche at an average age of 18.6 years, giving a recall period of 5.6 years, ranging from 1.4 to 10.4 years. While some studies benefit from continuous follow-up during adolescence, yielding shorter recall intervals (42), there is a gap in the literature concerning recall bias in menarcheal data within a few years after the event (43). The majority of studies affirm the validity of menarche recalled in adulthood (44).

The use of the QEPS model stands out as a methodological strength, allowing for a separation of the specific pubertal growth from the continuous basic growth during puberty. Compared to earlier models, QEPS provides a more individualized and precise view of growth patterns around menarche (16). The selection of the study population, limited to close-to-term-born, healthy individuals in Sweden with at least one Nordic-born parent, can be looked upon as a limitation by its homogeneity, affecting generalizability. However, this research was done with the purpose of providing information from a homogenous study population as a reference population. Thereafter, it will be possible to compare our findings with girls in other populations and ethnicities. Parental heights were also partly based on self-reported data, posing a theoretical limitation of the study. The GrowUp_1990_Gothenburg cohort used in this study constitutes an area representative of Sweden, encompassing a range of socioeconomic statuses, and the sample of 793 individuals in a community-based setting with longitudinal growth data enhances the generalizability of the findings. Collecting menarcheal data in close proximity to the event, at an average age of 18.6 years, further enriches the dataset (23).

### 4.1 Clinical relevance

This study shows a robust relationship between menarcheal age and growth at pubertal onset. At onset of the pubertal growth spurt, menarche occurred at mean 3.2 years later. Postmenarcheal height gain averaged 8 cm with a large variability from 0.2 to 31 cm and was strongly inversely correlated with age at menarche. This is of clinical importance since parents of girls with early menarche often are concerned of a future short adult stature. Our study shows that girls with early menarche (before 12 years of age) have a median height gain of 12.8 cm postmenarche, whereas girls with menarche above 14.2 years of age have a median height gain of just 3.1 cm after menarche. MPH is used worldwide as a target adult height. In this study, individuals were estimated to be taller than their MPH when menarche occurred later than 12.6 years of age.

### 4.2 Conclusion

This study significantly contributes to the understanding of menarcheal age and postmenarcheal growth. Multivariable analyses revealed that data available at pubertal onset could explain 44% of the variation in menarcheal age (range 8.2–17.2 years). The robust relationship between menarcheal age and growth at pubertal onset allows for early identification of individuals with abnormal growth. The diverse range seen in postmenarcheal growth, from 0.2 to 31.1 cm, has seldom been emphasized previously.

These findings hold important implications for clinical practice and future research, underscoring the importance of a personalized approach for growth monitoring in pediatric outpatient clinics. This research examines pubertal growth and menarcheal age using group-level data, while providing insights at the individual level. Here we used an optimally growing healthy population. Future research may focus on epidemiologic population-based studies of other ethnicities and further explorations at the individual level to develop predictive models for individual outcomes.

## Data Availability

The datasets presented in this article are not readily available, but the generated datasets can be provided upon reasonable request. However, the total raw dataset cannot be provided due to Swedish law. Requests to access the datasets should be directed to anton.holmgren@regionhalland.se.
